# Social Assistance Payments and Food Insecurity in Australia: Evidence from the Household Expenditure Survey

**DOI:** 10.3390/ijerph16030455

**Published:** 2019-02-04

**Authors:** Jeromey B. Temple, Sue Booth, Christina M. Pollard

**Affiliations:** 1 Demography and Ageing Unit, Melbourne School of Population and Global Health, University of Melbourne, Melbourne 3010, Australia; 2 College of Medicine and Public Health, Flinders University, Adelaide 5000, Australia; sue.booth@flinders.edu.au; 3 Faculty of Health Sciences, School of Public Health, Curtin University, Perth 6102, Australia; C.Pollard@curtin.edu.au

**Keywords:** food insecurity, access to food, social assistance payments, social security, Newstart allowance

## Abstract

It is widely understood that households with low economic resources and poor labour market attachment are at considerable risk of food insecurity in Australia. However, little is known about variations in food insecurity by receipt of specific classes of social assistance payments that are made through the social security system. Using newly released data from the 2016 Household Expenditure Survey, this paper reports on variations in food insecurity prevalence across a range of payment types. We further investigated measures of financial wellbeing reported by food-insecure households in receipt of social assistance payments. Results showed that individuals in receipt of Newstart allowance (11%), Austudy/Abstudy (14%), the Disability Support Pension (12%), the Carer Payment (11%) and the Parenting Payment (9%) were at significantly higher risk of food insecurity compared to those in receipt of the Age Pension (<1%) or no payment at all (1.3%). Results further indicated that food-insecure households in receipt of social assistance payments endured significant financial stress, with a large proportion co-currently experiencing “fuel” or “energy” poverty. Our results support calls by a range of Australian non-government organisations, politicians, and academics for a comprehensive review of the Australian social security system.

## 1. Introduction

In Australia, conservative estimates show food insecurity attributable to financial constraints is experienced by 4–5% of the population, with the rate significantly higher among Aboriginal and Torres Strait Islander people [1,2,3]. Addressing food insecurity in high-income countries such as Australia is important because of the deleterious consequences of exposure for individual health and wellbeing. A substantial and growing evidence base shows food insecurity is associated with symptoms of depression and anxiety, multimorbidity, lower levels of self-reported health status, poor nutrition, a greater likelihood of reporting social isolation, long-standing health problems and activity limitations, and a greater likelihood of reporting heart disease, diabetes, high blood pressure, or peripheral arterial disease [4,5,6,7,8,9,10,11,12,13]. Food insecurity experienced within households also has implications for the intergenerational transmission of health issues for children living in food-insecure households [14,15,16] and may also contribute to ongoing economic inequality [17]. Given these significant outcomes of food insecurity, many high-income countries have extensive social welfare safety nets to alleviate poverty which, in turn, reduces food insecurity at a population level. 

However, studies have shown that welfare reforms over recent years have had a severe impact on vulnerable populations and increased the likelihood of food insecurity. For example, in the UK, increased sanctioning of unemployment claimants led to an increase in the rate of adults attending food banks [18]. In the U.S., welfare reforms limiting access to immigrant populations had the impact of significantly increasing levels of food insecurity [19]. In Australia, too, evidence from a recent qualitative study showed changes to welfare eligibility by low-income single parents increased the risk of food insecurity [20].

Australian studies have also shown significantly higher levels of food insecurity among the unemployed relative to other Australians [2,21]. Indeed, numerous Australian studies have underscored the strength of economic factors (e.g., income and labour force status) in explaining exposure to food insecurity. Temple’s (2008) nationally representative study of food insecurity in Australia concludes that because of this strong association, policies must target improvements to economic wellbeing through revisiting the appropriateness of extant unemployment benefits and labour market programs [2]. 

Omitted from existing research on food insecurity and social assistance in Australia is an understanding of how the likelihood of food insecurity differs across the range of social assistance payments provided by the Federal Government. In this paper, newly released data from the 2016 ABS Household Expenditure Survey were used to investigate levels of food insecurity and financial wellbeing reported by recipients of a range of social assistance payments, broadly categorised as the Age Pension, Disability and Carer payments, Family Support payments, and Unemployment and Student allowances. 

### Background to Social Assistance Payments in Australia

The Australian social security system is intended to increase the wellbeing of the population by redistributing Government revenue collected in the tax system to individuals and families [22]. It is a broader part of a social protection system that includes direct expenditure on services and infrastructure (such as health, education, and community services), the superannuation system—which complements the age pension in Australia’s retirement income system—and payments, services, and investment to promote the efficient and effective functioning of the economy, which underpins individual and national wellbeing [22].

Relative to other Organisation for Economic Co-Operation and Development (OECD) member countries, Australia’s social security system is unique as (1) most social assistance cash payments are flat-rate entitlements funded by direct Government revenue, and (2) most benefits are heavily income- or asset-tested, with payment reducing as individual private resources increase [23]. This design enables Australia to have a relatively broad social safety net encompassing unemployment benefits and universal health care and assistance for vulnerable populations across the life course [24]. Concerns have been raised, however, about the erosion of the safety net and the particularly low levels of income support provided through social assistance payments, such as the Newstart Allowance—the key payment available for unemployed people of working age [25,26,27].

Previous Australian studies on food insecurity have focused on particularly vulnerable populations, many with an increased higher likelihood of receipt of some form of social benefit payments—for example, homeless or at-risk youth [28,29], students [30,31], refugees [32,33], Aboriginal and Torres Strait Islander peoples [3,34], older Australians [35,36] and those living in disadvantaged suburbs [37]. Despite this significant evidence base, there is a paucity of studies examining variations in food insecurity across a range of social assistance payments. This is important as variations in the prevalence of food insecurity by payment type may uncover populations at particular risk, which could be addressed through the existing social welfare system. 

In this study, we examine food insecurity by receipt of social assistance payments, broadly classified at the household level as the Age Pension, Disability and Carer payments, Family Support payments, Unemployment and Student allowances, and other Government pensions and allowances. At the individual level, we further analyse food insecurity by a number of specific social assistance payments. Among those discussed in this paper include [38]:

- Austudy: Available to persons aged 25 and over undertaking study or a full-time Australian apprenticeship. Basic rates start from $445 per fortnight for a single person with no dependent children.
- Abstudy: Available to persons of Aboriginal or Torres Strait Islander descent, undertaking an approved course on full-time Australian apprenticeship. Basic rates start from $445.80 per fortnight for a single person with no dependent children.
- Age pension: Available to persons aged 65 or over (if born before July 1952) to 67 and over (if born January 1957 and later). Basic rates start from $834.40 per fortnight for a single person. Subject to income and assets test.
- Carer payment: Available to persons providing constant care to 1 or more persons with a disability as determined by specific assessment tools and as a result of the carer role do not work. Basic rates start from $834.40 per fortnight for a single person. Subject to income and assets test.
- Disability support pension (DSP): Available to persons aged 16 or over, but less than Age Pension age, with a disability as defined by an impairment table, and who are unable to work or undertake training within the next two years. Basic rates start from $572.90 per fortnight for a single person (Independent).
- Newstart allowance: Available to Australian residents who are aged 22 or over (but less than age pension age) and unemployed. Basic rates range from $550 per fortnight for a single person depending on circumstances.
- Parenting payment: Available for parents who have a child under 6 (if partnered), or 8 (if single). Once the child is beyond these ages, the parent must enter into a job plan. Subject to stringent income and assets test. Basic rates of up to a maximum of $768 per fortnight, inclusive of a pension supplement.
- Youth allowance: Available to full-time students and Australian apprentices aged 16–24. Basic rates range from $244 to $768 per fortnight depending on household circumstances.

## 2. Materials and Methods 

### 2.1. Survey Data

Data for this study were from the Household Expenditure Survey (HES) conducted by the Australian Bureau of Statistics (ABS) over the period July 2015 to June 2016. The purpose of the HES was to “facilitate the analysis and monitoring of the social and economic welfare or Australian residents in private dwellings. The main users are government and other social and economic analysts involved in the development, implementation and evaluation of social and economic policies” [39].

The HES is a repeated cross-section design, with nine surveys conducted since 1974–1975. Since 2003–2004, the HES sample was drawn alongside respondents of the ABS Survey of Income and Housing (SIH). Of the 17,768 households recorded in the SIH, 10,046 were included in the HES. Dwellings were sampled using a stratified, multistage cluster design across a 12-month enumeration period to account for seasonality effects on income and expenditure.

As the HES samples private dwellings, a number of populations are excluded from our analyses. These include persons residing in hotels, boarding schools, and institutions. Also excluded are households containing members of non-Australian Defence forces, diplomatic personnel as well as households in very remote areas of Australia. Apart from houses and flats, the ABS consider persons residing in caravans, garages, tents, and other structures used as residences to be private dwellings. 

These data were collected by Australia’s official statistical agency, and accordingly, the protection of participants and the provision of data to us is enshrined in legislation. Specifically, data for the Household Expenditure Survey were collected by the ABS under the provisions of the *Census and Statistics Act (CSA) 1905*. Prior to field operations, the survey was submitted to the Australian Privacy Commissioner and tabled in the Australian Parliament. The confidentiality of these data is guaranteed under the Act and information was provided freely from respondents. Confidentialised data were made available to the authors for this study through the ABS and Universities Australia agreement. 

### 2.2. Measurement

The measures of food insecurity used by the ABS have heretofore been confined to measures of financial attributions of running out of food. For example, two item questions in the National Nutrition Survey and National Health Survey ask: “In the past 12 months were there any time(s) when you ran out of food and couldn’t afford to buy any more”. Those who reported yes to this question are considered food-insecure. The measure used in the HES is comparable but is likely to identify a more at-risk group of food-insecure persons [3]. Respondents in the 2009/10 HES were asked: “Over the past year, have any of the following happened to (you/your household) because of a shortage of money?” Those reporting ‘yes’ to ‘went without meals’ are coded as food-insecure. 

The HES also included a number of measures of financial wellbeing, consisting of measures of financial stress, income management, standard of living, and access to emergency funds. These measures provide a complimentary view of the financial position of food-insecure households in receipt of social assistance payments. Respondents were sought to identify whether in the previous 12 months, they had undertaken a number of financial stress behaviours, including seeking help from welfare or community organisations, pawning or selling something, seeking financial help from family or friends, or inability to heat their home or pay utility or other bills on time. As a summary measure of self-assessed financial wellbeing, respondents were further asked: “Thinking of your household’s situation over the last 12 months, which of the following statements best describes your financial situation?” A prompt card was then displayed listing: Spend more money than we get, just break even most weeks or able to save money most weeks. Furthermore, respondents were prompted: “Which of these statements best describes your household’s standard of living compared to 2 years ago?” A prompt card was then shown listing: Better than 2 years ago, the same as 2 years ago or worse than 2 years ago.

Finally, as a measure of financial resilience to unanticipated events, respondents were asked: “If all of a sudden your household had to get two thousand dollars for something important, could the money be obtained within a week”? Following a response, using a prompt card, respondents were asked to nominate the sources of the emergency funds from a list including: Savings, loan from bank/building society, loan from finance company, loan on credit card, loan from family or friends, loan from welfare or community organisation, sell something or from any other source.

In this descriptive study, we calculated the weighted prevalence of food insecurity by payment type with tests of proportions between groups. 

## 3. Results

Table 1 cross-tabulates source of household income and main source of social assistance payments by food insecurity status. The first panel of Table 1 displays the proportion of each group (food-secure by receipt of benefits and food-insecure by receipt of benefits) by the main source of household income. In the second panel, the broad social assistance payment types are tabulated by food security status.

Approximately 80% of Australian households who report food insecurity received some form of social assistance payment in 2015–2016 (82.4%), with 75% of food-insecure households in receipt of social assistance benefits listing this as the main source of household income (74.8%). Food-insecure households receiving social assistance payments are predominately in receipt of Disability and Carer payments (38%) and Unemployment and Student allowances (28.7%)—Table 1. By contrast, food-secure households in receipt of social assistance payments are more likely to receive the Age Pension (36.6%), with less than 10% being in receipt of Unemployment and Student allowances. Approximately 20% of food-insecure and 24% of food-secure households are in receipt of Family benefits. Of households not in receipt of social assistance payments, almost 90% of both food-insecure and -secure households receive wages from employment. Approximately 2.8% of households reported food insecurity (as measured by going without a meal due to financial constraints).

As indicators of financial wellbeing, the HES includes a number of measures of financial stress (Appendix Table A1), income management, standard of living (Appendix Table A2), and access to emergency funds (Appendix Table A3). About 60% of food-insecure households in receipt of social assistance payments reported seeking financial help from friends or family and about 43% had sought assistance from a welfare or community organisation (Appendix Table A1). Sixty per cent could not pay utility bills on time, about 35% had pawned or sold something, and 30% reported being unable to heat their home. Less than one per cent of households who are food-secure and not in receipt of social assistance payments were unable to heat their home or had pawned something, and <6% had difficulty paying for utilities. Almost half of food-insecure households receiving social assistance payments reported spending more money than they receive and just over half reported their standard of living as worse than 2 years ago (Appendix Table A2). By contrast, 82% of food-secure households not receiving benefits reported their standard of living as the same or better than two years ago and 60% of this group were able to save money most weeks.

Seventy three percent of food-insecure households in receipt of social assistance payments could not raise $2000 within a week, with very few options from capital markets with respect to raising funds (Appendix Table A3). The key source of emergency funds for this group was reported as loans from family or friends (20%). By contrast, only 6% of food-secure households with no social assistance payments and 16% of those with social assistance payments could not raise emergency funds, with a much broader range of emergency fund sources across capital markets and personal resources.

When these measures of financial stress (Appendix Table A1), income management, and standard of living (Appendix Table A2) and access to emergency funds (Appendix Table A3) are cross-tabulated by social assistance type, households in receipt of Disability and Carer payments as well as Unemployment and Student allowances are shown to be in a financially precarious position. In comparison, among social assistance recipients, households in receipt of the Age Pension appear to have lower levels of financial stress, higher self-assessed standard of living, and an improved access to emergency funds.

The specific social assistance benefit received by individuals who are members of food-insecure households in the HES is shown in Table 2. The higher prevalence of food insecurity reported by those receiving Unemployment, Student, and Disability payments is highlighted in these data. Prevalence was highest among people receiving Austudy/Abstudy (14%), Disability Support Pension (12%), Newstart Allowance (11%) and the Carer payment (11%). Age pension recipients were significantly less likely to report food insecurity (<1%), as were those receiving the DVA Disability pension.

## 4. Discussion

International evidence shows that individuals in receipt of social assistance payments are at increased risk of food insecurity [40]. To date, there has been scant evidence on the prevalence of food insecurity by social assistance payment type in Australia. Of the information available, a 2013 study of people accessing Anglicare Australia’s emergency relief centres in two states reported that 31% of food-insecure households were reliant upon the Newstart allowance and 44% on the disability support pension [41]. Using nationally representative data, this study confirms the significantly higher prevalence of food insecurity among recipients of Australian government social assistance payments—with about 80% of households reporting food insecurity receiving some form of social assistance payment. 

Particularly high levels of food insecurity were found among households in receipt of Unemployment, Student, Carer, and Disability payments, suggesting the inadequacy of these transfers. Specifically, when examined at the level of specific payment types, individuals in receipt of Newstart Allowance (11%), Austudy/Abstudy (14%), Disability Support Pension (12%), the Carer Payment (11%), and Parenting Payment (9%) were at significantly higher risk of food insecurity compared to those in receipt of the Age Pension (<1%) or no payment (1.3%). 

In 2018, the Australian Prime Minister indicated that his Government prioritises an increase to the Age Pension above any changes to the Newstart Allowance [42]. This is despite research underscoring the deleterious financial position of those in receipt of unemployment and student payments. For example, the Newstart Allowance has long been criticised for not providing a healthy living allowance, and the problem has compounded over time due to the method of indexation [26,27]. 

The current study findings are consistent with research showing that the standard of living experienced by older Australians has increased considerably over the past decade, with higher levels of income and wealth relative to previous generations of older persons [43,44,45]. The basic rate for the Age Pension is currently AUD $834 per fortnight compared with AUD $550 per fortnight for Newstart Allowance recipients. Further, the Australian Council of Social Services (2018) reported that the poverty gaps (the average depth of poverty for those living below the poverty line) among people aged 65 years and over in income support households were much lower than those across the whole population [45]. The mismatch between indicated government policy for older and younger and working age people and research evidence is concerning.

Apart from Newstart Allowance recipients, the higher levels of food insecurity reported by those in receipt of Disability Support Pension are consistent with recent research on disabilities, health conditions, and food insecurity in Australia and internationally [46,47]. Temple (2018) found that the onset of serious disability (OR 2.3 *p* < 0.01) or mental illness (OR 2.9 *p* < 0.001) more than doubled the odds of experiencing food insecurity in Australia [21]. Although the Disability Support Pension has a higher basic rate of payment than the Newstart Allowance, almost one in five food-insecure respondents in this current study are in receipt of the disability support pension. The findings are consistent with UK research, which shows that households with a disability are almost three times more likely to be foodbank users [48]. 

This study also identified those on Parenting and Carer payments were at an increased risk of food insecurity. These findings resonate with previous Australian research that found single parents were more likely to experience food insecurity due to factors such as income and housing instability [49,50]. Australia shifted its welfare policy context to ‘Welfare to Work’ in 2006, founded on the principle of mutual obligation where recipients must complete compulsory activities in order to access income support. Those receiving parenting benefits were transitioned to the lower-rate Newstart Allowance [50,51]. Single mothers relying on the Newstart Allowance experienced a struggle to buy basics such as food, reliance on foodbanks, and keeping children home from school as they were unable to provide food which met the school lunchbox policy [50]. The higher prevalence of food insecurity among persons in receipt of the Carer payment is consistent with recent evidence showing financial support is the greatest unmet need reported by Australian carers [52]. 

Our findings pointing to the higher prevalence of food insecurity on these payments is concerning given recent research on intergenerational transfer of disadvantage. Cobb-Clark (2017) has shown that households in receipt of Disability, Carer, and Parenting payments are at a strong risk of intergenerational persistence of disadvantage [53]. Of major concern is that children living in households dependent on these specific payments are more likely themselves to receive more intensive social assistance payments in their early adulthood and more likely to experience unemployment.

Finally, our findings underscore the deleterious financial position experienced by food-insecure households and those on specific social assistance payments in Australia. The high levels of ‘fuel or energy poverty’ faced by food-insecure Australians is of particular concern. About 30% of food-insecure households in receipt of social assistance payments reported being unable to heat their home, and 60% were unable to pay their utility bills on time. 

UK and U.S. research has also drawn attention to the relationship between food insecurity and fuel or energy poverty. Anderson et al. (2012) described the experience of ‘cold’ homes in the UK where households faced with financial difficulty cut the range and quality of food while simultaneously cutting energy consumption [54]. Large reductions in food expenditure have been reported in low-income households during colder than expected winter conditions [55]. Poor families living in the US reduced their food expenditure commensurate to increases in fuel expenditures when cold-weather shocks occurred, suggesting that existing social programs were ineffectual in buffering against these shocks [56]. Canadian evidence shows energy price shocks at the turn of the century led to an increase in the population at risk of food insecurity [57]. 

Australia has experienced significant energy price inflation following the deregulation of energy markets [58]. The high levels of concurrent energy poverty facing the food-insecure can lead to further financial burden, for example, the cost of reconnection or default payments [59]. Australian households that were disconnected or at risk of disconnection experienced very difficult financial circumstances, in which they often struggled to afford necessities such as food and housing [59]. In a recent article, Nelson et al. (2019) suggested, among other solutions, increasing income support for particular groups (including those on Newstart) as well as the reform of state-based energy concessions to combat energy poverty [60]. 

These solutions, by reducing energy costs and increasing income support, would undoubtedly reduce the likelihood of vulnerable populations experiencing food insecurity. International evidence suggests that increases to social assistance payments reduce the prevalence and severity of food insecurity at a population level. For example, in Newfoundland and Labrador in Canada, the prevalence of food insecurity reduced dramatically from 2007–2011 due to welfare reforms [61]. Another Canadian study found that a one-off increase in social assistance benefits led to a significant decline in moderate and severe food insecurity among households on social assistance [62].

### Study Limitations

In interpreting results from this study, it is important to recognise the limitations. Firstly, the measure of food insecurity in Australia comprising of measures of ‘going without meals due to financial constraints’ captures neither temporality nor severity [2]. However, currently, these are the only population-based measures available. Our study, however, does raise the question of the use of household expenditure data to improve measurement and understanding of food insecurity. Future research on this issue is currently underway by the authors.

The measurement issue is also important given the differences in food insecurity prevalence experienced by those of working age or younger populations compared to older persons in receipt of the age pension. Previous studies have note that food insecurity attributable to financial constraints tends to decrease in older age in Australia [2,35]. Part of this may reflect a measurement issue. Herein, we focus only on financially attributable food insecurity, but international studies show that storage, transportation, and functional barriers are all important in explaining food insecurity in older populations [63]. Thus, we are likely to be biasing downward the prevalence of food insecurity among older Australians. Moreover, the prevalence of food insecurity may be higher for age pension recipients who rent rather than own their home. Secondly, there is the role of selective mortality in these cross-sectional data. As individuals with higher economic and social resources are more likely to exhibit higher survival prospects relative to their financially disadvantaged peers, in cross-sectional data we may be observing these individuals in later life.

More generally, the HES data are cross-sectional, and it is not possible to draw any type of causal relationship between receipt of certain payments and food insecurity. Specifically, we do not know if prior to receipt of certain payments, they were food-insecure, or only insecure once on payments. However, recent evidence shows that experience of involuntary job loss (OR 2.6 *p* < 0.001) or difficulty finding employment (OR 2.5 *p* < 0.001) within the past 12 months increases the odds of food insecurity by about 2.5 times [21]. The purpose of this paper has been to present prevalence rates of food insecurity across a range of social benefit payment types. Further multivariable analyses, ideally with longitudinal data, should be conducted to provide further detail on the experiences of food insecurity faced by social assistance payment recipients in Australia.

## 5. Conclusions

This is the first Australian study to examine the differences in the prevalence of food insecurity across a wide range of social assistance payments. We found a high prevalence of food insecurity among those receiving Australian Government social assistance payments, including the Newstart Allowance, Austudy/Abstudy, Disability Support Pension, the Carer payment, and Parenting payment. The relatively higher levels of income support through the Age Pension payment may have had a protective effect on food insecurity and financial wellbeing, demonstrating the benefits of addressing income inadequacy that has been found in the international literature. Due to differences in indexing the respective payments, the level of the Newstart Allowance as a percentage of the age pension has fallen from 90% in the 1990s to 60% today [64].

Australian advocates for action to reduce poverty and inequality have called for the Government to ‘raise the rate’ of Newstart and related payments, noting that Newstart has not increased in real terms for 24 years [65]. Recent Australian modelling indicates that an increase in the Newstart Allowance to $800 per fortnight in Australia would significantly decrease the poverty gap in Australia by about 11% [66]. Our results support calls by a range of Australian non-government organisations, politicians, and academics calling for a comprehensive review of the Australian social security system [67]. Our findings, when combined with others in the Australian literature, suggest well designed increases in the Newstart, Disability, Student, Carer, and disability payments may improve the material resources of food-insecure households and thus ameliorate their food insecurity experience and potentially offset health and economic risks [4,5,6,7,8,9,10,11,12,13,14,15,16,17].

## Figures and Tables

**Table 1:** Food insecurity and receipt of social assistance, by main source of income and source of social assistance—households, weighted (%), 2016.

Food-Secure:	Yes		No ^1^	
Receipt of Social Assistance Benefits:	No	Yes	No	Yes
**Main Source of Income** ^2^				
Employee Income	85.7	43.2	89.4	23.8 ***
Own Business Income	4.8	3.5	6.8	0.4 ***
Government Pensions & Allowances	0.0	41.3	0.0	74.8 ***
Other Income	9.5	12.1	3.8	1.0 ***
**Main Source of Social Assistance Payments** ^3^				
No Social Assistance	100	n.a.	100	n.a.
Age Pension	n.a.	36.6	n.a.	9.4 ***
Disability and Carer Payments	n.a.	12.2	n.a.	38.3 ***
Family Support Payments	n.a.	24.1	n.a.	19.7
Unemployment and Student Allowances	n.a.	9.7	n.a.	28.7 ***
Other Government Pensions/Allowances	n.a.	17.4	n.a.	4.0 ***
**Unweighted *n*** ^4^	3855	5884	38	263
**Weighted %** ^5^	43.5	53.7	0.5	2.3

^1^ Going without meals due to financial constraints in the previous 12 months; ^2^ Household main source of income in the previous 12 months; ^3^ Source of social assistance benefits at the household level; n.a. not applicable for households not in receipt of social assistance payments; ^4^ number of raw observations; ^5^ percentages weighted using survey weights to account for non-response. *** *p* < 0.001 for test of proportions. Test of proportions conducted between each social assistance benefit groups. That is, assistance benefit recipients (food-secure) compared with assistance benefit recipients (food-insecure) and for non-assistance benefits also (insignificant differences).

**Table 2:** Food insecurity prevalence and percentage receiving social assistance payments—persons, weighted (%), 2016.

Social Assistance Benefit Type	Food Insecurity (%) ^3^	Food-Insecure in Receipt of Benefit (%) ^2^	*n*^1^ =
Austudy/Abstudy	13.8 **	3.9 *	83
Age Pension	<1 ***	4.6 ***	3733
Carer Allowance	5.0 **	4.8 **	470
Carer Payment	10.9 ***	5.8 ***	255
Carer Supplement	5.9 **	6.9 **	565
Disability Pension (DVA)	<1	<1	101
Disability Support Pension	12.4 ***	18.9 ***	803
Family Tax Benefits	5.5 ***	17.3 ***	1284
Newstart Allowance	11.0 ***	14.6 ***	645
Parenting Payment	9.0 ***	5.7 ***	322
Youth Allowance	6.0 *	4.0 *	233
Any Social Assistance Payment?			
Yes	3.9	64.2	8545
No	1.3	35.8	10,660

^1^ Unweighted sample size per benefit; ^2^ percentage of food-insecure persons in receipt of each social assistance payment. Tests of proportions for proportion of food-insecure in receipt of each benefit compared to food-secure in receipt of each benefit; ^3^ food insecurity prevalence. Tests of proportions for in receipt of each payment compared to those not in receipt; percentages weighted using survey weights to account for non-response; *** *p* < 0.001 ** *p* < 0.01 * *p* < 0.05 denoting significance tests for tests of proportions.

## Appendix A

**Table A1:** Indicators of Financial Stress (%) by Food Insecurity Status and Receipt of Social Assistance, Households 2015/2016.

	Food Insecure					Main Source of Household Social Assistance Benefits in Cash				
Receives Social Assistance Benefits:	No		Yes		None	Age	Disability and	Family Support	Unemployment and	Other Pensions
	No	Yes	No	Yes		Pension	Carer Payments	Payments	Student Allowances	and Allowances
Sought assistance from welfare/community organisation	<1	2.7	1.1	42.7	<1	1.6	10.4	5.4	9.6	<1
Pawned or sold something	<1	2.3	12.9	34.7	1.1	<1	7.8	5.2	8.8	1
Sought financial help from friends or family	3.9	6.9	57.6	59.7	4.5	2.5	14.2	14	21.5	4.2
Unable to heat home	<1	2.1	22.8	30.4	1	1.9	6.9	2.6	8.3	1.1
Could not pay gas/electricity/telephone	5.6	10.5	55.9	59.3	6.1	4	19.6	22	20.9	6.1
Could not pay registration / insurance on time	2.3	3.8	35.2	31.6	2.7	1	6.7	8.6	12.1	2.3
Unweighted (n)	3855	5884	38	263	3894	2680	836	1177	528	926
Weighted (%)	43.5	53.7	0.5	2.3	44.0	19.9	7.4	13.4	5.9	9.5

**Table A2:** Management of Household Income and Standard of Living (%) by Food Insecurity Status and Receipt of Social Assistance, Households 2015/2016.

	Food Insecure					Main Source of Household Social Assistance Benefits in Cash				
Receives Social Assistance Benefits:	No		Yes		None	Age	Disability and	Family Support	Unemployment and	Other Pensions
	No	Yes	No	Yes		Pension	Carer Payments	Payments	Student Allowances	and Allowances
*Management of Household Income*										
Spend more money than we get	10	13.6	22	46.1	10.1	8.9	21	19.8	20.9	12.3
Just break even most weeks	33.2	49.6	60.2	48.8	33.5	49.5	50.1	52.3	55.1	41.1
Able to save money most weeks	56.8	36.8	17.8	5.1	56.4	14.6	28.9	27.4	24	46.6
*Present Standard of Living*										
Better than 2 years ago	41	22.3	15.2	22.9	40.7	13.1	22.9	31.6	27.9	25.1
The same as 2 years ago	40.9	50.6	32.5	24.9	40.8	62.6	43.6	39.3	30.6	50.6
Worse than 2 years ago	18.1	27.1	52.4	52.1	18.5	23.4	33.5	29.1	41.5	24.4
Unweighted (n)	3855	5884	38	263	3894	2680	836	1177	528	926
Weighted (%)	43.5	53.7	0.5	2.3	44.0	19.9	7.4	13.4	5.9	9.5

**Table A3:** Access to Emergency Funds (%) by Food Insecurity and Receipt of Social Assistance, Households 2015/2016.

	Food Insecure					Main Source of Household Social Assistance Benefits in Cash				
Receives Social Assistance Benefits:	No		Yes		None	Age	Disability and	Family Support	Unemployment and	Other Pensions
	No	Yes	No	Yes		Pension	Carer Payments	Payments	Student Allowances	and Allowances
*Access to Emergency Funds*										
Could not raise $2000 within a week	6.3	16.1	32.5	73.2	6.6	11.3	34.7	21.5	33.9	6.7
*Source(s) of Emergency Funds*										
Own Savings	77.9	65.1	19	10.4	77.3	74.5	45.4	51.9	41.9	78.6
Loan from a Bank, Building Society	14.3	9.9	8.9	<1	14.3	5.8	9.3	13.3	9	12.4
Loan from a Finance Company	4.3	1.8	0	1.1	4.2	<1	2.6	2.3	1.2	3.3
Loan on Credit Card	19.5	11.8	25.1	2.2	19.6	8.1	9.5	15.9	11.2	13.4
Loan from Family or Friends	19.4	15.9	26.7	19.7	19.5	8.5	15.3	25.3	20.5	16.6
Loan from Welfare or Community Organisation	<1	<1	<1	<1	<1	<1	2.4	1	<1	<1
Sell Something	9.1	4.9	6.7	4.3	9.1	1.7	3.8	7.8	7.1	6.7
Other Sources	2.4	2.8	3.1	<1	2.5	2.7	1.9	2.3	3.9	3.1
Unweighted (n)	3855	5884	38	263	3894	2680	836	1177	528	926
Weighted (%)	43.5	53.7	0.5	2.3	44.0	19.9	7.4	13.4	5.9	9.5
